# Clinical pharmacy in Israel versus the US, can we "compare apples to apples"?—commentary

**DOI:** 10.1186/s13584-021-00508-3

**Published:** 2021-12-13

**Authors:** Eyal Schwartzberg, Eli Marom

**Affiliations:** 1 School of Pharmacy, Community Clinical Pharmacy and Regulatory Management MSc Program, Ben Gurion University, Beer Sheva, Israel; 2 Pharmaceutical and Enforcement Division, Ministry of Health, Jerusalem, Israel; 3 Professional Committee, Pharmaceutical Society, Tel Aviv, Israel

**Keywords:** Clinical pharmacy, Comparison, Healthcare systems, Israel, US

## Abstract

Clinical pharmacy is an umbrella of pharmaceutical services that is practiced by clinical pharmacists. Clinical pharmacists improve the quality of drug therapy, minimize the risk for drug related problems, reduce the risk of morbidity and mortality associated with polypharmacy and decrease the overall healthcare expenditure. Consequently, clinical pharmacy is focused on both the needs of the individual patient, as well as of the healthcare system. Clinical pharmacy is now well-established and practiced worldwide and in the last two decades has been implemented successfully in Israel. This commentary maintains that the comparison of clinical pharmacy practice in Israel and in the United States, published by AJ Rose et al., has several limitations that need to be considered when devising a road map that will fit the Israeli health care system and its environment. Emphasis should be placed on the implementation of automation and robotics, promulgating regulations to allow for integration of pharmacy technicians, and allocating funds for such services.

## Main text/Discussion

In their comprehensive manuscript, ‘Seven key parameters that facilitate clinical pharmacy practice: comparison between Israel and the United States’, Adam J Rose and his colleagues [1] suggest a road map for the advancement of clinical pharmacy in Israel. They define seven key parameters that, if implemented, may contribute to the realization of clinical pharmacy as a profession.

In this commentary we demonstrate that these elements have, to a large extent, already been implemented in Israel. We also contend that there are significant differences between the Israeli and the American healthcare systems that we believe should have been discussed to a greater extent by the authors. Arguably, such differences may have led the authors to perceive “clinical pharmacy” services as less developed in Israel.

Table 1 summarizes current “real world data” about the situation of clinical pharmacy in the US and Israel, with regards to the seven parameters explored in the discussed manuscript. It should be noted that although there are differences in the number and scope of pharmacists practicing clinical pharmacy in Israel, they are continually increasing in number and gaining in importance in the Israeli healthcare landscape.

**Table 1:** Comparison of 7 parameters of clinical pharmacy practice US vs. Israel, real world data

Parameter	US [1]	Isarel
1. Advance Degree, which is compatible with the skills required for the practice of clinical pharmacy	Pharm D	Pharm D—Hebrew University MSc (since 2008), Community Clinical Pharmacy & Regulatory Management—Ben Gurion University (Since 2019)
2. Residency: The existence of postgraduate residency slots for pharmacists which are specifically intended to allow them to acquire and refine the skills necessary to practice	Yes	Hands-on training, continuing education courses. Internal training—No formal continuous academic education
3. Credentialing: recognition of clinical pharmacy as a specialty within the pharmacy profession	Board certified pharmacotherapy and other specialty– 1/8 of all pharmacists in the US	No board certification but acknowledged as a specialty by the Ministry of Health in its definition of clinical pharmacy in 2002, and by job descriptions, depending on the employer: civil service and HMOs [2, 3]
4. Supply: having enough clinical pharmacists to perform the work demanded of them	Continuous residency programs—approximately 5000 every year	Two programs aiming to meet the demand, with between 40 and 50 graduates every year (MSc and Pharm D)
5. Scope of practice: a. Legal permission to practice semi-independently in a meaningful way b. Prescribing medications c. Ordering & interpreting labs & taking medication history d. Legal permission to bill	Practiced in the US	a. Currently most of the practice is under HMOs, while independent practice is legally allowed b. Dependent prescribing medications is allowed to all clinical pharmacists. Few medications are allowed to be prescribed with no prior physician authorizations c. Dependent on internal protocols per institution [4] d. When practicing privately, a pharmacist can bill
6. Demand: demand by employers to hire and utilized clinical pharmacists fully	Practiced in the US	Such demand exists and is growing steadily, from handful at the beginning of the millennia to approximately 150 in 2021. (Currently all HMOs in Israel employ clinical pharmacists, as do most government owned hospitals, HMOs owned hospitals and some privately owned hospitals). Clinical pharmacists are also employed as QPPVs (qualified person for pharmacovigilance) in pharmaceutical companies
7. Acceptance of the clinical pharmacist role by other medical professionals as part of the healthcare team	Practiced in the US	The clinical pharmacist role was acknowledged in the MOH director general circular issued to all organizations within the Israeli health system. [3]

One of the key elements which makes a direct comparison between these systems difficult, is the fact that the healthcare landscape of each system is very different. We believe this is imperative to take into consideration when trying to compare clinical services. These differences are prominent in all aspects of healthcare, structure, scale, and scope of services available to citizens.

In Israel, the national health insurance law covers every citizen. It offers full and highly accessible medical services to all. The situation in the US is rather different and although the level of medicine and pharmacy is advanced, there is a great deal of variability in the availability of such services to different socio-economic groups. In Israel, the level of private medicine is rather low when compared to the US, as is the average expenditure per capita: $2786 and $10,586 respectively [5]. Additionally, in Israel all patients’ medical information is digitalized. This allows for faster and more accurate control and flow of information between different organizations and primary, secondary, and tertiary care. This in turn may lead to superior clinical results and fewer drug related problems.

It should also be remembered that Israel is a relatively young country, and this influences the development of its academic pharmacy programs, as well as the scope of pharmacy education and practice. Important changes to the pharmacy ordinance, which allowed pharmacists to intervene and consult clinically, have been in effect for only the last 10 years, while in the US such changes have existed for more than 40 years.

In addition to the regulatory landscape, another important difference relates to graduate and post-graduate learning. Until the beginning of this millennia, there was only one School of Pharmacy in Israel (that of The Hebrew University in Jerusalem) and pharmacy education concentrated mainly on the product, rather than on the patients. The opening of a second school at the Ben Gurion University introduced vast changes in the educational bachelors’ program in both schools, making them increasingly practice and clinically oriented. With the change in MSc in clinical pharmacy to the PharmD program at the Hebrew university in 2008, and the opening of the MSC in community clinical pharmacy and regulatory management program at the Ben Gurion University in 2019, another pillar of pharmacy practice was introduced and implemented. Consequently, this increased the number of practicing clinical pharmacist in Israel.

The number of pharmacists varies vastly between the two countries, with 315,000 pharmacists in the US compared with only 9100 in Israel [6, 7]. Although the difference between the number of practicing pharmacists per population (95 per 100,000 population in the US versus 76 in Israel), is not staggering, the number of clinical pharmacists with clinical accreditation in the States is approximately 1/8 (12.5%) of the total number of all pharmacists, whereas in Israel only 150 clinical pharmacists are practicing (1.6%) [1, 8].

We believe that the following should be taken into consideration when comparing the two clinical pharmacy landscapes. Subsequently they need to be addressed by the Israeli Ministry of Health, academia, employers, and healthcare organizations in Israel, if clinical pharmacy is to evolve effectively and comprehensively in Israel.

- *Utilization and implementation of automation in pharmacies in the US*: This enables pharmacists to dedicate most of their working hours to patients, their careers and interacting with other healthcare practitioners. This concept of automation and dispensing robots is widely accepted and practiced in the United States, whereas in Israel it is only in its early days. The ability to integrate such systems will free pharmacists’ time to move from traditional dispensing into more clinical activities. To promote automation in Israel, the MOH should develop comprehensive guidelines for the implementation of such services. Potential users, HMOs, and private employers should assess the feasibility of such solutions. Additionally, financial support/incentives should be offered by the MOH to organizations implementing such solutions in conjunction with clinical pharmacy services. Such incentives are also known as "support tests" and are published periodically by the MOH for diverse health challenges the government wishes to promote.
- *Legislation, reimbursement for clinical services such as medication therapy management (MTM) and medication reconciliation under Medicare part D*” In the United States, Medicare (the governmental health insurance program for the elderly) reimburses pharmacists for providing MTM services. Additionally, Medicare and certain private health insurance companies pay for hospitalizations of their beneficiaries using a diagnosis-related group (DRG) payment system. If a hospital can effectively treat you for less money than Medicare pays for your DRG, then the hospital makes money on that hospitalization. If the hospital spends more money caring for you than Medicare gives it for your DRG, then the hospital loses money on that hospitalization. Thus, there is an incentive for hospitals to employ specialists who will decrease expenditure and maximize revenue. Such strategy may include employing clinical pharmacists who have been demonstrated to decrease the risk of drug related problems in hospitalized patients and consequently decrease costs associated with hospital stays. In Israel, reimbursement for all pharmacy services (including clinical pharmacy) is currently included in the national health insurance law. If the situation is to change in Israel, one will need distinct legislation regarding DRG and the role of clinical pharmacists in this respect. Thus, clinical pharmacy should be defined as an expert and separate pharmaceutical service that is compensated accordingly.
- *Pharmacy technicians*—in the United States, the law allows for pharmacy technicians to practice. Currently, there are approximately 450 000 technicians, thus for every pharmacist there is an additional one and a half technician [9]. In Israel currently, only pharmacists are allowed to practice pharmacy. As the number of pharmacists to citizens per capita is similar between Israel and the USA, the workload, and especially dispensing, is more intense in Israel. This in turn does not allow time for additional activities to be practiced, including clinical pharmacy. The MOH, along with the Ministry of Finance should promote legislation that will allow certification and establishment of the pharmacy technician role. In addition, they should provide the funds required to employ them. Such funds should be supported by a robust pharmaco-economic model, measuring how re-routing pharmacists from traditional logistical activity into clinical pharmacy improves patients’ outcomes, thereby reducing healthcare costs and subsequently demonstrating return of investment.

## Conclusion

Despite the above-mentioned challenges, one should also consider the achievements of clinical pharmacy in Israel thus far. Over the last 10 years, most healthcare providers in Israel adopted and implemented clinical pharmacy services, and this shift continues. As the pharmacy profession continues to evolve, so will clinical pharmacy services. Therefore, it will need to adapt to the changing environment and concentrate on the aging population, polypharmacy, and medication adherence for chronic patients, as well as the implementation of tele-medicine advancements, which will fortify brick and mortar services [10].

Such changes will require additional legislation with clear definition of the role and scope of interventions of clinical pharmacists. To complement this, there will be a need for pharmacy technicians to allow pharmacists to engage in clinical activities.

Making such changes will require the provision and publication of data on hard outcomes regarding the feasibility and contribution of clinical pharmacy in Israel. Currently most of the evidence regarding the viability of clinical pharmacy services comes from the international arena. However, when presenting to local stakeholders and decision makers in Israel, it is advisable to use local evidence-based data that was created and published in the Israeli health ecosystem and thus, avoid the “NIH” phrase (Not Invented Here). The outcomes measures used in the creation of such data should be agreed prior to their implementation with key opinion leaders in the Ministries of Health and Finance as well as with the HMOs. This will allow the result to be transformed into a practical action plan.

Finally, there is an old saying in Hebrew: "If there is no flour, there is no Torah”, which means that there is no learning without an effort or substance. Therefore, if stakeholders deem clinical pharmacy/pharmacists important for improving the quality of patient care and wish to develop and implement such services, they will need to reimburse and allocate adequate funds for this important process.

## Data Availability

Not applicable.
